# Effect of regional versus general anesthesia on thirty-day outcomes following carotid endarterectomy: a cohort study

**DOI:** 10.1097/JS9.0000000000000356

**Published:** 2023-04-14

**Authors:** Leigh A. Kline, Venkatraman Kothandaraman, Ziyad O. Knio, Zhiyi Zuo

**Affiliations:** a Department of Anesthesiology, University of Virginia Health; b School of Medicine, University of Virginia, Charlottesville, Virginia, USA

**Keywords:** carotid endarterectomy, general anesthesia, major morbidity, mortality, perioperative outcomes, regional anesthesia

## Abstract

The effect of regional versus general anesthesia on carotid endarterectomy outcomes is debated. This study assesses the effect of anesthetic technique on major morbidity and mortality and additional secondary endpoints following carotid endarterectomy.

**Materials and methods:**

This was a retrospective propensity-matched-cohort analysis investigating elective carotid endarterectomy patients in the 2015–2019 American College of Surgeons National Surgical Quality Improvement Program (*n*=37 204). The primary endpoint was 30-day mortality and major morbidity, defined as stroke, myocardial infarction, or death. Secondary endpoints included minor morbidity, bleeding events, healthcare resource utilization, and length of hospital stay. Univariate, multivariable, and survival analyses were applied.

**Results:**

The 1 : 1 propensity-matched-cohort included 8304 patients (4152 in each group). Regional anesthesia was associated with similar incidences of major morbidity and mortality [odds ratio (OR), 0.81 (95% CI, 0.61–1.09); *P* = 0.162] and unplanned resource utilization [OR, 0.93 (95% CI, 0.78–1.11); *P* = 0.443], but lower incidences of minor morbidity [OR, 0.60 (95% CI, 0.44–0.81); *P* < 0.001] and bleeding events [OR, 0.49 (95% CI, 0.30–0.78); *P* = 0.002], and a shorter length of hospital stay [1.4 vs. 1.6 days; mean difference, -0.16 days (95% CI, -0.25 to -0.07); *P* < 0.001]. On multivariable analysis, regional anesthesia remained independently predictive of minor morbidity [adjusted odds ratio (AOR), 0.58 (95% CI, 0.42–0.79); *P* = 0.001] and bleeding events [AOR, 0.49 (95% CI, 0.30–0.77); *P* = 0.003]. Significance was maintained on survival analysis for these two endpoints. A mortality benefit was observed on univariate [OR, 0.50 (95% CI, 0.25–1.00); *P* = 0.045], multivariable [AOR, 0.49 (95% CI, 0.24–0.96); *P* = 0.043], and survival analysis (*P* = 0.045).

**Conclusions:**

Carotid endarterectomy patients receiving regional anesthesia experience favorable outcomes compared to propensity-matched general anesthesia controls.

## Graphical abstract

**Figure:**
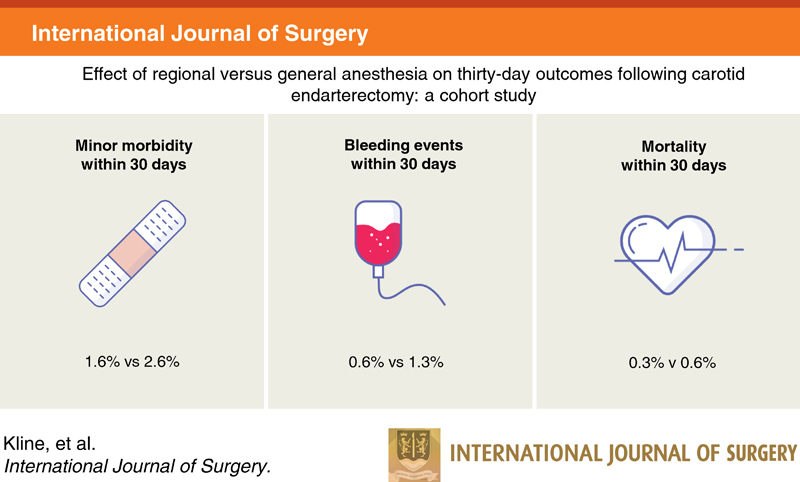

## Introduction

Highlights

- The effect of regional versus general anesthesia on carotid endarterectomy complications remains unclear.
- A matched-cohort study design may reduce the effect of confounding by anesthetic technique.
- Regional anesthesia is associated with similar incidences of 30-day major morbidity and mortality and unplanned resource utilization.
- Regional anesthesia is associated with fewer minor morbidity and bleeding events.
- Robust fragility indices support the above findings.

The optimal anesthetic technique for carotid endarterectomy remains highly debated^1^. One advantage of regional anesthesia is that it allows for the ‘gold standard’ awake neurologic assessment for perioperative ischemia and hypoxia^2^–^6^. Other proposed benefits include better hemodynamic stability, decreased vasopressor use, and preserved cerebrovascular reflexes that maintain cerebral perfusion and reduce the need for a shunt (which may damage the arterial wall and lead to embolism)^4^,^7^–^9^. Conversely, disadvantages of regional anesthesia include the need for a patient who is comfortable and compliant with akinesia. Additionally, regional anesthesia offers limited airway protection and may require a technically difficult intraoperative emergent tracheal intubation^4^,^6^. The advantages of general anesthesia include a secure airway and a more comfortable operating environment for both the surgeon and the patient^4^,^6^. On the other hand, disadvantages of general anesthesia include hemodynamic instability and increased usage of shunts during cross-clamping due to less effective neuromonitoring^5^,^10^–^14^.

The GALA Trial Collaborative Group (2008) demonstrated no difference in primary outcome (a composite of 30-day stroke, myocardial infarction (MI), or death) between the two anesthetic techniques in a landmark randomized controlled trial^3^. While these results have been replicated by some studies^12^,^15^–^18^, others have demonstrated a clinical benefit with regional anesthesia^10^,^11^,^19^–^24^. Several studies found an increased risk for postoperative MI with general anesthesia^19^–^21^. Furthermore, a few studies found that patients undergoing general anesthesia had higher rates of strokes and transient ischemic attacks^10^,^11^,^22^.

Given the discordant conclusions of various studies, further investigation into the impact of anesthetic technique is warranted. Furthermore, robust methodology must be employed in order to balance cohorts, as selection bias often results in regional anesthesia cohorts comprising individuals of better functional status^25^.

This retrospective study was designed to investigate whether anesthetic technique was associated with major morbidity and mortality (stroke, MI, or death within 30 days) following elective carotid endarterectomy in a propensity-matched-cohort sample. The goal of this methodology was to reduce confounding by indication for primary anesthetic technique. Secondary endpoints that were investigated included minor morbidity, bleeding events, unplanned healthcare resource utilization, and length of hospital stay. The authors hypothesized that the use of regional anesthesia during carotid endarterectomy would be associated with lower incidences of major morbidity and mortality, minor morbidity, bleeding events, unplanned resource utilization, and a shorter length of hospital stay.

## Material and methods

### Study design and data sources

This research was retrospectively registered with clinicaltrials.gov (https://clinicaltrials.gov/ct2/show/NCT05706688). The study was exempt from Institutional Review Board approval given that it was a retrospective cohort analysis of a national de-identified database. The American College of Surgeons National Surgeons Quality Improvement Program (ACS-NSQIP) database from 2015 to 2019 was queried, with carotid endarterectomy defined by the Current Procedural Terminology code 35301. The work has been reported in line with the Strengthening the Reporting of Cohort Studies in Surgery (STROCSS) Criteria^26^, Supplemental Digital Content 1, http://links.lww.com/JS9/A330.

### Patient inclusion and exclusion criteria

After the initial query, the following criteria were applied in order to produce a more homogenous investigation sample. Only elective, nonemergent cases were included. Patients with concurrent procedures were excluded. Patients with preoperative documentation of acute kidney injury, end-stage renal disease, metastatic disease, wound infection, and sepsis were excluded. Patients with American Society of Anesthesiologist Physical Status (ASA) classification 5 were excluded. These criteria were agreed upon by the study authors *a priori*. General anesthesia cases were identified by ‘General’ documentation in primary anesthesia type, without any further specifications in secondary anesthesia type. Regional anesthesia cases were identified by any combination of ‘Local,’ ‘MAC/IV Sedation,’ and ‘Regional’ documentation in the primary anesthesia type, without the appearance of ‘General’ in the secondary anesthesia type.

### Measurements and data handling

The primary independent variable was anesthetic technique (regional vs. general). Independent variables also included age, sex, BMI, hypertension, insulin-dependent diabetes, current smoker, chronic obstructive pulmonary disease (COPD), congestive heart failure (CHF), chronic steroid use, functional status, and the American Society of Anesthesiologists (ASA) physical status classification (ASA 4 versus ASA 1-3).

The primary endpoint was major morbidity and mortality, defined as stroke, MI, or death within 30 days.

Secondary endpoints included minor morbidity, bleeding events requiring transfusion, unplanned resource utilization, and length of hospital stay. Minor morbidity included reintubation, prolonged (>48 h) ventilator dependence, pneumonia, deep venous thrombosis, pulmonary embolism, superficial surgical site infection (SSI), deep incisional SSI, organ space SSI, wound dehiscence, sepsis, septic shock, acute kidney injury, and progressive renal insufficiency. Unplanned resource utilization included unplanned readmission and unplanned reoperation.

### Statistical analysis

Statistical analysis was performed with R version 4.2.0 (R Core Team)^27^. Regional anesthesia cases were identified, and general anesthesia cases were matched using propensity scores computed by patient demographics and medical comorbidities. Specifically, cases were matched by the following variables: age, sex, BMI, hypertension, insulin-dependent diabetes, current smoker, COPD, CHF, chronic steroid use, functional status, and ASA physical status classification (ASA 4 versus ASA 1-3). A 1:1 nearest neighbor propensity score matching algorithm using probit regression covariate estimates without replacement was applied. Balance among matched pairs was assessed by standardized mean differences^28^. Propensity score matching has been applied in prior studies; the proposed advantage of this methodology is adjusting for baseline differences between those receiving general and regional anesthesia^15^,^20^,^24^,^29^. Not infrequently, patients receiving regional anesthesia have fewer comorbidities^25^. Despite this, there remains the possibility for residual confounding between cohorts^30^.

The associations between anesthetic technique and composite endpoints or anesthetic technique and component outcomes were first assessed with univariate analyses. Pearson’s *χ* ^2^-test without continuity correction was applied to categorical variables while Student’s *t*-test was applied to the length of hospital stay. Additionally, odds ratios (OR), 95% CI, and fragility indices were calculated following *χ* ^2^ methodology^31^. The fragility index is a quantitative measure of how many subject-dependent classifications influence the statistical significance of a hypothesis test, with greater numbers suggesting a more robust difference between groups^32^,^33^.

Multivariable analyses were then performed on primary and secondary endpoints demonstrating statistical significance and a robust fragility index on univariate testing. Multiple logistic regression modeling was applied, with adjustments made for age, sex, BMI, hypertension, diabetes, smoking, COPD, CHF, chronic steroid use, functional status, ASA physical status classification, and anesthetic technique when significance was demonstrated on univariate testing at α less than or equal to 0.05. Only complete cases were considered, and missing data were not imputed. Subsequent variable selection was accomplished by backward stepwise model adjustment by Akaike information criterion. Backward stepwise adjustment, in contrast to forward stepwise adjustment, was applied such that all predictors would be considered in the null model, essentially favoring more rather than fewer predictors in the final regression. Regardless, the Akaike information criterion assesses the overall model fit and favors parsimony, a balance between over- and under-fitting. Independent predictors were not added/eliminated stepwise by statistical significance but rather by their contribution to a more optimized adjusted model (as demonstrated by a lower Akaike information criterion)^34^,^35^. Adjusted odds ratios (AOR) and 95% CI are reported for independent predictors, and model discrimination was assessed with the c-statistic^36^.

Survival analyses were then performed on the same endpoints. Kaplan–Meier curves modeling days of event-free survival against anesthetic technique were constructed. The risk attributable to anesthetic technique was quantified with the log-rank *P-*value^37^,^38^

Continuous variables were summarized by mean (SD), while categorical variables were summarized by frequency (%). All hypothesis tests were two-sided, with significance defined by α less than or equal to 0.05.

## Results

**Figure 1:**
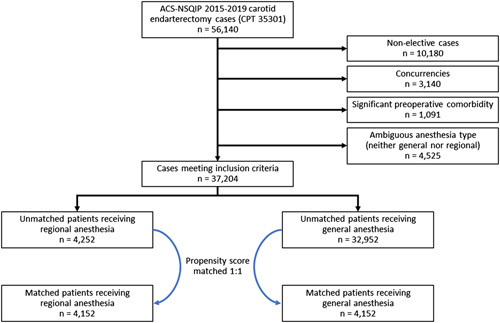
Consolidated Standards of Reporting Trials diagram detailing selection of patients within each cohort, including numbers of patients in each cohort before and after matching. ACS-NSQIP, American College of Surgeons National Surgical Quality Improvement Program.

**Figure 2:**
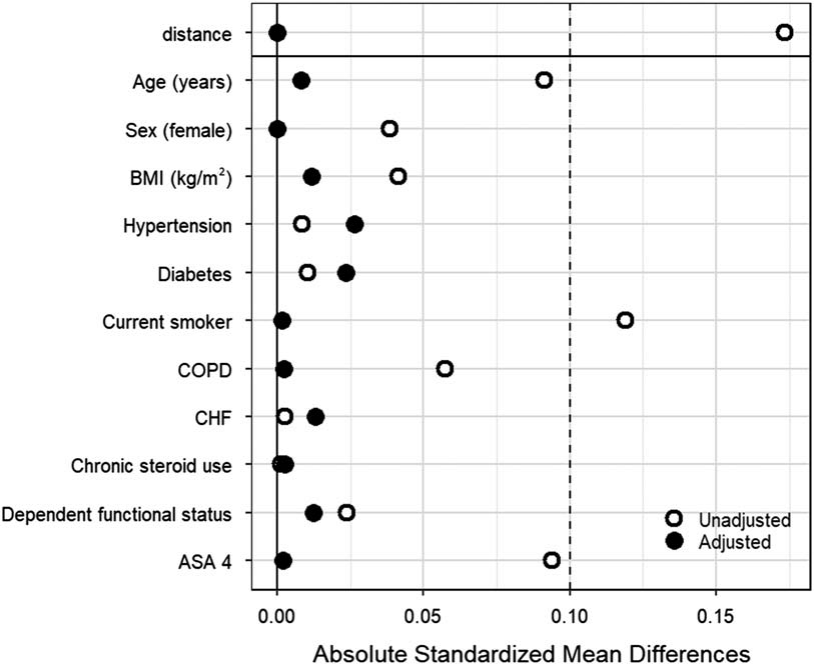
Absolute standardized mean differences before (*n*=37 204) and after (*n*=8304) matching the regional and general anesthesia cohorts 1:1 by propensity score. ASA, American Society of Anesthesiologists physical status; CHF, congestive heart failure; COPD, chronic obstructive pulmonary disease.

Of 37 204 carotid endarterectomy patients meeting inclusion criteria, 4252 (11.4%) received regional anesthesia. After 1:1 propensity score matching by patient demographics and medical comorbidities, a total of 8304 patients were matched (4152 in each study group) (Fig. 1). Balance between cohorts was achieved as evidenced by standardized mean differences < 0.1 (Fig. 2). The distribution of patient demographics and medical comorbidities in the regional and general anesthesia cohorts both before and after matching are presented in Table 1.

**Table 1:** Demographics and medical comorbidities by anesthetic technique, before and after propensity score matching.

	Before propensity score matching			After propensity score matching		
	No./total (%)		*P*	No./total (%)		*P*
Characteristic	General (*n*=32 952)	Regional (*n*=4252)		General (*n*=4152)	Regional (*n*=4152)	
Age, mean (SD), years	70.7 (8.7)	71.5 (8.7)	< 0.001	71.5 (8.7)	71.5 (8.7)	0.710
Female sex	13113/32952 (39.8%)	1624/4252 (38.2%)	0.045	1577/4152 (38.0%)	1577/4152 (38.0%)	> 0.999
BMI, mean (SD), kg/m^2^	28.9 (5.8)	28.7 (5.6)	0.005	28.6 (5.4)	28.7 (5.6)	0.585
Hypertension	27620/32952 (83.8%)	3574/4252 (84.1%)	0.694	3533/4152 (85.1%)	3493/4152 (84.1%)	0.224
Insulin-dependent diabetes	3889/32952 (11.8%)	481/4252 (11.3%)	0.351	446/4152 (10.7%)	477/4152 (11.5%)	0.279
Current smoker	9243/32952 (28.0%)	983/4252 (23.1%)	< 0.001	963/4152 (23.2%)	966/4152 (23.3%)	0.938
COPD history	3777/32952 (11.5%)	413/4252 (9.7%)	< 0.001	402/4152 (9.7%)	405/4152 (9.8%)	0.912
CHF history	412/32952 (1.3%)	53/4252 (1.2%)	0.983	44/4152 (1.1%)	50/4152 (1.2%)	0.534
Chronic steroid use	994/32952 (3.0%)	131/4252 (3.1%)	0.817	128/4152 (3.1%)	126/4152 (3.0%)	0.899
Dependent functional status	731/32802 (2.2%)	80/4244 (1.9%)	0.150	85/4152 (2.0%)	78/4152 (1.9%)	0.580
ASA 4	6304/32893 (19.2%)	662/4237 (15.6%)	< 0.001	652/4152 (15.7%)	649/4152 (15.6%)	0.928

ASA, American Society of Anesthesiologists; CHF, congestive heart failure; COPD, chronic obstructive lung disease.

On univariate analysis, regional anesthesia was not associated with rates of major morbidity and mortality [2.0% (85/4152) vs. 2.5% (104/4152); OR, 0.81 (95% CI, 0.61–1.09); *P*=0.162] or resource utilization events [6.1% (254/4152) vs. 6.5% (271/4152); OR, 0.93 (95% CI, 0.78–1.11); *P*=0.443]. However, regional anesthesia was associated with a lower incidence of minor morbidity [1.6% (65/4152) vs. 2.6% (108/4152); OR, 0.60 (95% CI, 0.44–0.81); *P*<0.001] and bleeding events [0.6% (26/4152) vs. 1.3% (53/4152); OR, 0.49 (95% CI, 0.30–0.78); *P*=0.002]. The fragility indices for these endpoints were robust. The length of hospital stay was shorter in the regional anesthesia cohort [1.4 vs. 1.6 days; mean difference, -0.16 days (95% CI, -0.25 to -0.07); *P*<0.001]. Of the component outcomes demonstrating a significant association with anesthesia type, thirty-day mortality was considered for subsequent analyses given that it is a clinically meaningful outcome demonstrating a lower incidence in the regional anesthesia cohort [0.3% (12/4152) vs. 0.6% (24/4152); OR, 0.50 (95% CI, 0.25–1.00); *P*=0.045] (Table 2).

**Table 2:** Univariate analyses by anesthetic technique (regional vs. general).

	No./total (%)				
Outcome	General (*n* = 4152)	Regional (*n* = 4152)	Odds ratio (95% CI)	*P*	Fragility index
Major morbidity and mortality (composite)	104/4152 (2.5%)	85/4152 (2.0%)	0.81 (0.61–1.09)	0.162	9
Stroke	59/4152 (1.4%)	57/4152 (1.4%)	0.97 (0.67–1.39)	0.852	19
Myocardial infarction	39/4152 (0.9%)	26/4152 (0.6%)	0.66 (0.40–1.09)	0.105	4
Mortality	24/4152 (0.6%)	12/4152 (0.3%)	0.50 (0.25–1.00)	0.045	1
Minor morbidity (composite)	108/4152 (2.6%)	65/4152 (1.6%)	0.60 (0.44–0.81)	< 0.001	16
Unplanned reintubation	44/4152 (1.1%)	20/4152 (0.5%)	0.45 (0.27–0.77)	0.003	7
Ventilator dependence >48h	20/4152 (0.5%)	12/4152 (0.3%)	0.60 (0.29–1.23)	0.156	4
Pneumonia	37/4152 (0.9%)	20/4152 (0.5%)	0.54 (0.31–0.93)	0.024	2
Deep venous thrombosis	1/4152 (0.0%)	2/4152 (0.0%)	2.00 (0.18–22.07)	0.564	5
Pulmonary embolism	3/4152 (0.1%)	3/4152 (0.1%)	1.00 (0.20–4.96)	> 0.999	6
Superficial SSI	12/4152 (0.3%)	15/4152 (0.4%)	1.25 (0.58–2.68)	0.563	7
Deep incisional SSI	6/4152 (0.1%)	4/4152 (0.1%)	0.67 (0.19–2.36)	0.527	4
Organ space SSI	4/4152 (0.1%)	2/4152 (0.0%)	0.50 (0.09–2.73)	0.414	4
Wound dehiscence	3/4152 (0.1%)	1/4152 (0.0%)	0.33 (0.03–3.20)	0.317	4
Sepsis	11/4152 (0.3%)	6/4152 (0.1%)	0.54 (0.20–1.47)	0.225	4
Septic shock	4/4152 (0.1%)	3/4152 (0.1%)	0.75 (0.17–3.35)	0.705	5
Acute kidney injury	3/4152 (0.1%)	3/4152 (0.1%)	1.00 (0.20–4.96)	> 0.999	6
Progressive renal insufficiency	4/4152 (0.1%)	1/4152 (0.0%)	0.25 (0.03–2.24)	0.180	3
Bleeding event	53/4152 (1.3%)	26/4152 (0.6%)	0.49 (0.30–0.78)	0.002	8
Unplanned resource utilization (composite)	271/4152 (6.5%)	254/4152 (6.1%)	0.93 (0.78–1.11)	0.443	27
Unplanned readmission	209/4152 (5.0%)	191/4152 (4.6%)	0.91 (0.74–1.11)	0.356	21
Unplanned reoperation	102/4152 (2.5%)	108/4152 (2.6%)	1.06 (0.81–1.39)	0.675	22
Length of hospital stay, mean (SD), days	1.6 (1.9)	1.4 (2.3)	−0.16 (−0.25 to −0.07)	< 0.001	NA

SSI, surgical site infection.

Regional anesthesia remained an independent predictor of minor morbidity [AOR, 0.58 (95% CI, 0.42–0.79); *P*=0.001], bleeding events [AOR, 0.49 (95% CI, 0.30–0.77); *P*=0.003], and mortality [AOR, 0.49 (95% CI, 0.24–0.96); *P*=0.043] after adjusting for covariates on multivariable analysis (Appendix A, Supplemental Digital Content 2, http://links.lww.com/JS9/A331).

Additional predictors of minor morbidity included insulin-dependent diabetes [AOR, 2.23 (95% CI, 1.52–3.22); *P*<0.001], current smoker [AOR, 1.71 (95% CI, 1.23–2.35); *P*=0.001], COPD [AOR, 2.13 (95% CI, 1.43–3.10); *P*<0.001], CHF [AOR, 2.99 (95% CI, 1.22–6.28); *P*=0.008], and dependent functional status [AOR, 2.14 (95% CI, 0.94–4.21, *P*=0.044] with model c-statistic=0.654. Additional predictors of bleeding events included age [AOR, 1.03 per year (95% CI, 1.01–1.06); *P*=0.014], BMI [AOR, 0.93 per kg/m^2^ (95% CI, 0.89–0.97); *P*=0.003], and ASA 4 [AOR, 1.97 (95% CI, 1.16–3.20); *P*=0.009], with model c-statistic=0.684. Additional predictors of mortality included age [AOR, 1.06 per year (95% CI, 1.02–1.11); *P*=0.004], COPD [AOR, 4.36 (95% CI, 2.08–8.67); *P*<0.001], and ASA 4 [AOR, 2.48 (95% CI, 1.20–4.89); *P*=0.010], with model c-statistic=0.758 (eTable 1, Supplemental Digital Content 3, http://links.lww.com/JS9/A332).

**Figure 3:**
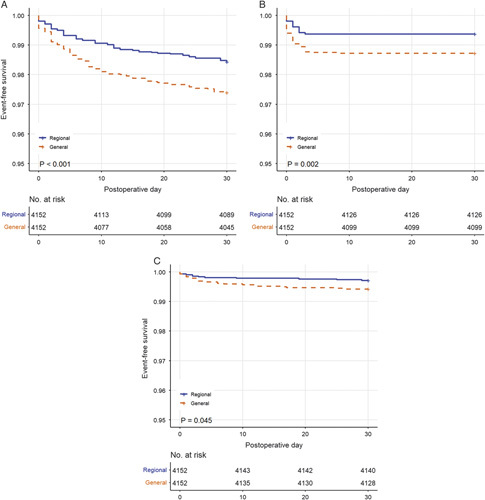
Survival analysis by anesthetic technique for (a) minor morbidity, (b) bleeding events, and (c) mortality.

Survival analysis demonstrated a significantly longer time to minor morbidity (*P*<0.001), bleeding events (*P*=0.002), and death (*P*=0.045) in patients receiving regional anesthesia (Fig. 3).

## Discussion

This study determined that patients receiving regional anesthesia had lower event rates of minor morbidity, bleeding requiring transfusion, and mortality within 30 days of elective carotid endarterectomy. Event rates for the composite primary outcome (stroke, MI, or death), wound complications, and unplanned resource utilization were comparable between the two anesthetic techniques. The length of hospital stay was statistically significantly shorter in the regional anesthesia cohort by 0.16 days, which may be of limited clinical significance. Regardless, there were no composite or component endpoints in which patients receiving regional anesthesia experienced worse outcomes than their propensity-matched general anesthesia counterparts.

The present study corroborates the conclusion by the GALA Trial Collaborative Group (2018) that major morbidity and mortality are not associated with anesthetic technique^3^. However, other clinically meaningful benefits with regional anesthesia were identified in the present study, and the component analysis demonstrates a lower 30-day mortality rate. It is possible that the nonsignificant difference in stroke and MI rates has skewed the results of the GALA Trial Collaborative Group (2018) to find no difference. Interestingly, Lumas *et al*.^24^ identified a signal in their composite, but not their component, outcomes in a matched-cohort study design.

There are other matched-cohort studies that found higher rates of MIs in carotid endarterectomy patients receiving general anesthesia^19^–^21^. One such study by Leichtle *et al*.^19^ determined that an elevated risk of postoperative MI was observed only in general anesthesia patients with preoperative neurologic symptoms; asymptomatic patients did not share this association. This finding could explain some of the discrepancies found between these studies. None of the matched-cohort studies conducted thus far have found a difference in the incidence of stroke postoperatively. Interestingly, a randomized prospective study by Orlicky *et al*.^39^ also found no difference in stroke incidence but found that patients receiving general anesthesia had more silent brain infarctions when 24 h preoperative and 24 h postoperative MRIs were compared to those of patients who received local anesthesia (*P*=0.031).

Malik *et al*.^29^ found no mortality difference in their 22 845 propensity-matched-cohort sample, but showed that general anesthesia was associated with a significantly higher rate of perioperative transfusions (*P*=0.037) and perioperative pneumonia (*P*=0.027), a finding shared by the present study. Their finding that patients with low hematocrit and low platelet count were more likely to get regional anesthesia supports the use of propensity-matching.

There are unmatched cohort studies that have identified a reduction in rates of stroke^10^,^11^,^22^, MI^23^, and mortality^40^, in carotid endarterectomy patients receiving regional anesthesia. However, these results may be influenced by selection bias, with confounding factors influencing the choice of anesthetic technique. Moreover, these results have not been reliably replicated in other unmatched cohort studies^12^,^16^–^18^. Another factor that could contribute to the varied results from these studies is the length of follow-up. An unmatched cohort study by Gürer *et al.* 10 was one of the only studies that examined long-term outcomes and found that at 8–10 year follow-up, there was no statistically significant difference in terms of restenosis-free survival, ipsilateral stroke-free survival, or overall survival.

The present study identified additional characteristics that were unequally distributed between the unmatched cohorts; the general anesthesia cohort before propensity score matching had an overrepresentation of patients who were younger, female, of lower BMI, current smokers, COPD patients, and ASA 4. Propensity score matching produced cohorts that were balanced in all of the aforementioned characteristics, with standardized mean differences less than 0.1. While propensity score matching reduces the effect of confounding, it does not eliminate the potential for selection bias^30^.

This study is significantly strengthened by reporting fragility indices for all outcomes, complementing the *P*-value with an interpretable measure of patient classification on statistical significance^32^,^33^. At a minimum, the classification of 16 minor morbidity events and 8 bleeding events would need to be changed to the opposite class to alter the statistical significance of these findings. Importantly, the significance of the mortality association with anesthetic technique is contingent on just one classification.

There are several limitations that are inherent to the study design. The endpoints and covariates that could be investigated in this retrospective observational study are limited to those captured by the ACS-NSQIP database. Endpoints such as patient-reported outcomes and covariates such as preoperative neurologic deficits are examples of variables not captured by the ACS-NSQIP database. Furthermore, ACS-NSQIP outcomes sampling is limited to 30 days postoperatively, thus stroke and mortality incidences beyond this time point cannot be computed. Also, there is the possibility that with such a large sample size, the study was over-powered to detect differences between the two cohorts. Thus, it is important to consider the reported odds ratios rather than rely solely on statistical significance to quantify the difference in outcomes between anesthetic techniques. While an increased risk of adverse events was observed in the general anesthesia cohort, a causal relationship could not be determined. A randomized clinical trial design would be better suited to test such a hypothesis. Further research is certainly warranted in order to explain the mechanisms by which regional anesthesia confers these clinical benefits.

## Conclusions

In summary, the present study demonstrates a favorable postoperative complication profile in carotid endarterectomy patients receiving regional anesthesia. A regional anesthetic technique is independently predictive of a reduction in minor morbidity, bleeding, and mortality events, even after adjusting for preoperative and operative characteristics.

## Ethical approval

This research was retrospectively registered with clinicaltrials.gov (https://clinicaltrials.gov/ct2/show/NCT05706688). The study was exempt from Institutional Review Board approval given that it was a retrospective cohort analysis of a national de-identified database. The data is in a publicly accessible database.

## Sources of funding

This study is supported by Robert M. Epstein Professorship from the University of Virginia, Charlottesville, VA. The funder has no role in experimental design, data analysis, manuscript writing, or decision on publication of this study.

## Conflicts of interest disclosure

The authors declare that they have no known competing financial interests or personal relationships that could have appeared to influence the work reported in this paper.

## Author contribution

L.A.K.: investigation, writing – original draft; V.K.: investigation, writing – original draft; Z.O.K.: methodology, software, formal analysis, writing – original draft; Z.Z.: conceptualization, resources, writing – review, and editing, supervision, project administration.

## Data availability statement

American College of Surgeons National Surgical Quality Improvement Program and the hospitals participating in the ACS-NSQIP are the source of the data used herein. The Participant Use Data File (PUF) is a Health Insurance Portability and Accountability Act (HIPAA)-compliant data file containing cases submitted to the American College of Surgeons National Surgical Quality Improvement Program®. To request a copy of the PUF, individuals (data recipients) must agree to comply with the terms and conditions set forth in the Data Use Agreement, provide contact information, and complete a short online questionnaire. Once the information provided by the data recipient is received and processed by ACS-NSQIP staff, a website address will be submitted electronically to the data recipient. The data recipient will then have 10 days (240 h) to visit the website and download the data file.

## Guarantor

The ACS-NSQIP database was accessed by Z.O.K. Z.O.K. and Z.Z. have full access to all the data in the study and take responsibility for the integrity of the data and the accuracy of the data analysis.

## Provenance and Peer Review

Not commissioned, externally peer-reviewed.

## Supplementary Material
