# Supplementary material for: Effect of regional versus general anesthesia on thirty-day outcomes following carotid endarterectomy: a cohort study
Source: Int J Surg. 2023 Apr 14;109(5):1291–8. doi: 10.1097/JS9.0000000000000356 (PMC10389611; doi:10.1097/JS9.0000000000000356)
Supplement: Supplementary file 2 [file js9-109-1291-s002.docx]

**Appendix A**

Multivariable analyses for minor morbidity, bleeding events, and mortality.

|  | Adjusted odds ratio (95% CI) | Adjusted *P* value |
| --- | --- | --- |
| Minor morbidity predictors, model c = 0.654 | | |
| Insulin-dependent diabetes | 2.23 (1.52 to 3.22) | < 0.001 |
| Current smoker | 1.71 (1.23 to 2.35) | 0.001 |
| COPD history | 2.13 (1.43 to 3.10) | < 0.001 |
| CHF history | 2.99 (1.22 to 6.28) | 0.008 |
| Dependent functional status | 2.14 (0.94 to 4.21) | 0.044 |
| Regional anesthesia | 0.58 (0.42 to 0.79) | 0.001 |
| Bleeding events predictors, model c = 0.684 | | |
| Age, years | 1.03 (1.01 to 1.06) | 0.014 |
| BMI, kg/m^2^ | 0.93 (0.89 to 0.97) | 0.003 |
| ASA 4 | 1.97 (1.16 to 3.20) | 0.009 |
| Regional anesthesia | 0.49 (0.30 to 0.77) | 0.003 |
| Mortality predictors, model c = 0.758 | | |
| Age, years | 1.06 [1.02 to 1.11] | 0.004 |
| COPD history | 4.36 [2.08 to 8.67] | < 0.001 |
| ASA 4 | 2.48 [1.20 to 4.89] | 0.010 |
| Regional anesthesia | 0.49 [0.24 to 0.96] | 0.043 |

Abbreviations: ASA, American Society of Anesthesiologists; BMI, body mass index; CHF, congestive heart failure; CI, confidence interval; COPD, chronic obstructive lung disease.
